# Brain Network Activation as a Novel Biomarker for the Return-to-Play Pathway Following Sport-Related Brain Injury

**DOI:** 10.3389/fneur.2015.00243

**Published:** 2015-11-20

**Authors:** Adam W. Kiefer, Kim Barber Foss, Amit Reches, Brooke Gadd, Michael Gordon, Ken Rushford, Ilan Laufer, Michal Weiss, Gregory D. Myer

**Affiliations:** ^1^Division of Sports Medicine, Cincinnati Children’s Hospital Medical Center, Cincinnati, OH, USA; ^2^Department of Pediatrics, University of Cincinnati College of Medicine, Cincinnati, OH, USA; ^3^Center for Cognition Action and Perception, Department of Psychology, University of Cincinnati, Cincinnati, OH, USA; ^4^ElMindA Ltd, Herzliya, Israel; ^5^St. Xavier High School, Cincinnati, OH, USA; ^6^Department of Orthopaedic Surgery, University of Cincinnati, Cincinnati, OH, USA; ^7^The Micheli Center for Sports Injury Prevention, Waltham, MA, USA; ^8^Sports Health and Performance Institute, The Ohio State University, Columbus, OH, USA

**Keywords:** brain network activation, EEG, concussion, hockey, return to play

## Abstract

Children and adolescent athletes are at a higher risk for concussion than adults, and also experience longer recovery times and increased associated symptoms. It has also recently been demonstrated that multiple, seemingly mild concussions may result in exacerbated and prolonged neurological deficits. Objective assessments and return-to-play criteria are needed to reduce risk and morbidity associated with concussive events in these populations. Recent research has pushed to study the use of electroencephalography as an objective measure of brain injury. In the present case study, we present a novel approach that examines event-related potentials via a brain network activation (BNA) analysis as a biomarker of concussion and recovery. Specifically, changes in BNA scores, as indexed through this approach, offer a potential indicator of neurological health as the BNA assessment qualitatively and quantitatively indexes the network dynamics associated with brain injury. Objective tools, such as these support accurate and efficient assessment of brain injury and may offer a useful step in categorizing the temporal and spatial changes in brain activity following concussive blows, as well as the functional connectivity of brain networks, associated with concussion.

## Introduction

Children and adolescents may be at a higher risk for concussion then adults due to an increase in brain plasticity and a decrease in barrier to the maturing cortex (1, 2). This susceptibility is compounded by longer recovery times and increased associated symptoms in children compared to adults (1). Early research identified concussions as transient injuries with close to 90% of patients having symptoms subside within 7–10 days after the injury occurred, but a few recent studies have revealed that long-term brain damage may actually take place following the initial injury (1, 3–5). It is no surprise, then, that after the initial injury, there is an increased risk of sustaining a second injury (6, 7) due to persistent, potentially unknown, associated neurophysiological deficits. Evidence also indicates that multiple, seemingly mild concussions may result in exacerbated and prolonged neurological deficits: the social and financial impact of which have yet to be adequately determined (8). Thus, the potential for exacerbated symptoms and poor long-term prognosis in youth athletes, strongly indicate that objective return-to-play (RTP) criteria are needed to reduce second concussion risk and morbidity associated with concussive events in pediatric populations.

Various diagnostic tools have been utilized for decades to determine the presence of a concussion in patients. Unfortunately, many of these diagnostic tools are subjective in nature and may lead to further issues for both doctors and patients involved, including misdiagnosis and premature RTP clearance (9). This increases the chances for further trauma and increased complications for the concussed patient (9–11), and, as a result, there is an impetus in the scientific community to identify straight-forward, objective, and efficient diagnostic tools for concussion assessment to accelerate treatment and recovery.

Recent research has pushed to study the use of electroencephalography (EEG) as an objective measure to determine the effects of concussion on the brain’s electrocortical activity due to its lower costs and its relatively low invasiveness compared to other methods of assessment. The usefulness of EEG, quantitative EEG (qEEG), and event-related potentials (ERPs) in concussion research (3, 12–16) has been demonstrated in recent years both in terms of its clinical value and in terms of its ability to elucidate the neural process associated with the effects of concussion and the recovery trajectory following injury (17, 18). Importantly, several studies have also shown dissociations between electrophysiological measures and clinical measures (12, 16). Unfortunately, concussion research in adolescents is relatively underexplored compared to that of adults (3, 12, 14, 19, 20), and because this population may be more susceptible to the effects of concussion due to the immaturity of the developing brain (20), there is now a growing interest in examining injuries and diagnostic biomarkers in this age group (21, 22).

One of the challenges for the utilization of EEG assessment in the adolescent population is due to the fact that the developing brain is a highly complex, multilayered system that is organized into very complicated interconnecting *neural networks* – unique local physical and functional connections between neurons. Thus, developing an understanding between underlying mechanisms driving neurological deficits through single electrode and single ERP-based analyses is extremely difficult, and also a current limitation in the field. New approaches that take into account the simultaneous cooperative function of local neural networks that operate together as larger scale, global networks are necessary for the adoption of EEG techniques as a widely adopted diagnostic tool in these populations.

A recent approach that examines ERPs via a brain network activation (BNA) analysis may provide the next step in the evolution of EEG as an accepted tool for diagnosis and recovery assessment. A detailed description of the BNA algorithm is provided elsewhere (23–27); thus, only a brief summary of the method is given here. BNA analysis is a new tool for evaluating the network dynamics associated with brain responses (i.e., ERPs). BNA analysis is performed on the ERP data recorded at each electrode location by clustering the basic time–frequency characteristics of the ERPs (i.e., waveforms at specific location, amplitude, frequency, and timing/latency), and finding the relations between the clusters. The BNA analysis involves two independent processes: a group level pattern recognition process used to generate the characteristic group’s network [Reference Brain Network Model (RBNM)] through a normative database made up of a separate group of subjects, and a single subject level similarity evaluation process. In the first stage, the algorithm seeks a state-unique multi-sited spatiotemporal pattern – the BNA group network (i.e., the RBNM). Specifically, the BNA algorithm extracts the interparticipant sequential temporal co-occurrence of pairs of ERP peaks (event pairs) extracted from signals that were band-pass filtered into different frequency ranges and that emerged in different spatial locations. Next, BNA integrates group-common event pairs of specific temporal relations, spatial locations frequencies, and amplitudes into a unified functional network, which is the BNA group network (25–27). This stage must be completed only once as it becomes the normative group for comparison for future clinical comparisons.

When considering patient prognosis, in the second stage of the analysis process, a BNA score is computed for each patient individually, which reflects the degree of similarity between the activity of the single subject and the normative RBNM. A BNA score of 0 indicates no similarity between the individual’s BNA pattern and the RBNM, whereas a BNA score of 100 represents complete similarity. Since the BNA score is a similarity score, a low BNA score implies that the patient deviates from the norm and that their brain functioning is impaired in certain cognitive aspects relative to the normative population. Therefore, a low BNA score might indicate the presence of a cognitive malfunction (26) Thus, the RBNM serves as a task-specific benchmark for the identification of potential changes over time in BNA score progressions of evaluated patients. The pattern represented by the RBNM constitutes a functional brain network, and BNA depicts the evolving network dynamics as a set of nodes connected by a set of links.

## Case

The current case was a 15-year-old male varsity high school hockey player who participated in a prospective research study on concussion incidence. Prior to the start of the season, he participated in a neurocognitive EEG assessment, as part of a larger testing battery. Within 36 h of this initial testing session, he sustained a head injury (November 14) during a preseason game. The injury occurred when the Case was skating behind his net in pursuit of the puck and was checked into the boards. The opponent hit the Case on his left side and the opponent’s elbow made contact with the left side of the Case’s head, forcing the right side of his head into the glass. He maintained consciousness, and skated back to the bench under his own power while holding his head with his hands. The Case did not report any associated amnesia, but did report headache, dizziness, photophobia, and tinnitus. A standardized assessment of concussion (SAC) sideline test was administered and consisted of orientation questions (month, day, location, and activity), memory recall (word list memory), concentration (counting numbers and listing months backwards), and neurological screening (checking strength, sensation, pupil response, coordination, balance and eye tracking). The Case did not complete the assessment to the satisfaction of the medical staff, and thus was not cleared for further participation for the remainder of the game and was not allowed to participate in scrimmage the following day.

## Pre-Concussion Visits/Landmarks

### Visit 1 – 36 h Prior to Concussion

The case performed a baseline neurocognitive EEG assessment 36 h prior to the injury, as part of a larger battery of tests.

## Post-Concussion Visits/Landmarks

### Visit 2 – 18 h Post Concussion

The Case performed the same neurocognitive EEG assessment within 18 h of the initial injury, as part of a larger battery of tests. These additional tests were not clinically standardized tests but rather exploratory measures of performance and thus were not used for clinical decisions. The subject also underwent assessment by a study-specific athletic trainer who reviewed the initial symptoms. The athlete stated that the initial symptoms had resolved except for a minor headache. The subject was asymptomatic for any subsequent testing sessions.

### Landmark 1 – 3–8 Days Post Concussion

This landmark was not a laboratory visit as part of the research study but instead was part of the standard clinical care of the athlete and took place at the athlete’s school. For the landmark time point, clinical decisions made were not performed by the research team nor did the research team discuss these decisions with the case. In order to RTP, the athlete followed the high school’s stepwise RTP protocol. The subject underwent complete physical and cognitive rest over the weekend. Upon return to school, he reported to the school athletic trainers and was symptom free at that point (3 days post injury). Based on the follow-up exam and the subsequent absence of all symptoms, the subject began his activity progression and was monitored for any return of symptoms. The standard protocol for RTP consists of daily increases in activity beginning with light aerobic exercise, sport specific exercise (i.e., skating), non-contact training drills, full contact training, and concludes with return to game competition. If any symptoms return, the athlete is downgraded to the previous step. The subject progressed through the RTP without incident and returned to play 11/22/2014 (8 days post injury). The neurocognitive EEG assessment was not considered in the medical clearance decision process and the EEG assessment was not administered at this time point.

### Visit 3 – 21 Days Post Concussion

The Case was symptom-free and performed the same neurocognitive EEG assessment only during this visit.

### Visit 4 – 50 Days Post Concussion

The Case performed the same neurocognitive EEG assessment, as part of a larger battery of tests. This visit was a normally scheduled mid-season assessment, as part of the larger clinical trial and the Case was symptom free.

### Visit 5 – 116 Days Post Concussion

The Case was symptom free and, again, performed the same neurocognitive EEG assessment, as part of a larger battery of tests. This visit was a normally scheduled postseason assessment, as part of the larger clinical trial.

## Method

**Figure 1:**
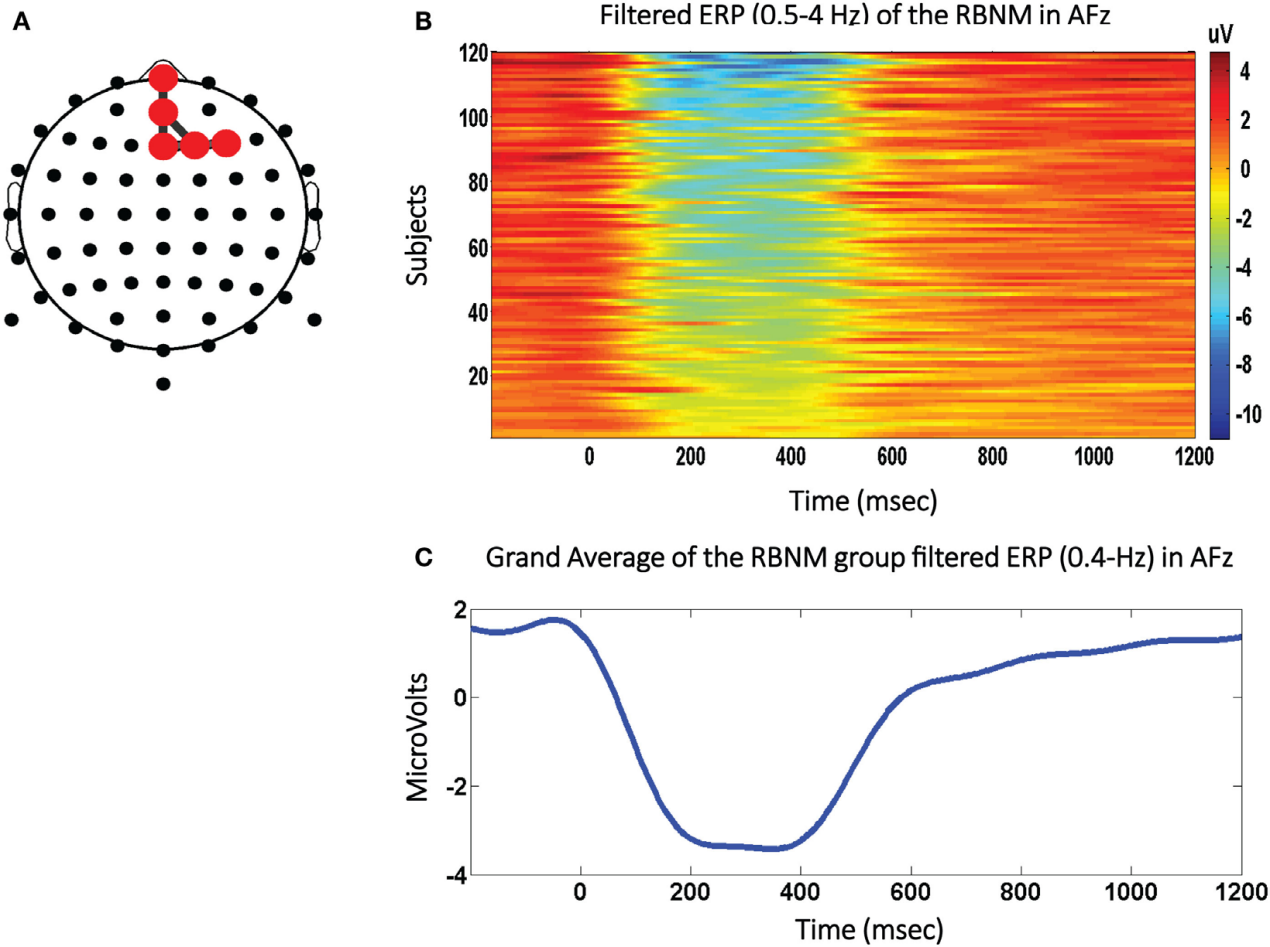
**The negative shift biomarker**. **(A)** The Reference Brain Network Model (RBNM) is displayed on the right side. The red dots represent delta band activations recorded from fronto-midline electrodes (FPz, AFz, and Fz) and from electrodes on the right side (F2 and F4). **(B)** The colored panel depicts individual peak activations recorded from each of 120 subjects included in the RBNM. **(C)** The delta band-passed grand average (120 subjects). The negative peak was most prominent during the ~200–400-ms interval observed at the electrodes included in the RBNM **(A)**.

The RBNM utilized in this study was generated from EEG waveforms recorded from healthy subjects (*N* = 120; 20 females; mean age = 14.58 years; range: 13–16 years; SD = 0.66) elicited to the frequent stimulus embedded within an auditory oddball task (28). See Figure 1A for the RBNM and the individual peak amplitudes (one blue dot per subject) recorded from an exemplar frontal electrode (Fpz; Figure 1B) along with the time-running grand average (120 subjects; Figure 1C). In Figure 1B, each row presents the ERP of each subject in the delta band for the duration of the recorded epoch by a color scale. Cool and warm colors represent negative and positive values, respectively. It can be observed that the negativity in the ERP waveform (Figure 1C) overlaps the blue segment embedded within the colored panel (Figure 1B). Each red dot in the RBNM represents activation recorded from a single channel in the delta frequency band (Figure 1A). Taken together, it can be observed that the RBNM consisted of a smooth negativity and a peak that was most prominent during the ~200–400 ms interval observed at fronto-midline electrodes (FPz, AFz, and Fz) and at electrodes on the right side (F2 and F4). This negativity is related to the negative shift phenomenon that is enhanced as a function of age (see Discussion).

Another independent large group of healthy participants (hereafter, the “test–retest group”) age matched to the RBNM Group (*N* = 41, 14 females; mean age = 15.03 years; range: 14–16 years, SD = 0.60) was recruited to calculate one SD interval of physiological variability defined by test–retest data. All subjects completed two consecutive BNA tests (*M* = 19 ± 36.80 days). Specifically, the range of normative data is defined based on the overall SD of the difference between two consecutive visits (SD = 30.10) computed on data gathered from the “test–retest” group. The 1 SD criterion was used to define a clinically meaningful change in BNA score between two visits and to allow detection of deviancy in the subject’s score from the accepted range of random fluctuations in the BNA score over time.

### Ethics Statement

This study was reviewed and approved by the Cincinnati Children’s Hospital Medical Center Institutional Review Board.

## Assessment

The current case performed two auditory-based cognitive tests: (1) an auditory oddball task and (2) an auditory go-no go test, but only data from the oddball task was analyzed in the current case. During this task, sounds are presented at an average rate of 1 every 1.5 s. A total of 80% of the sounds are 2000 Hz tones that repeat in frequency and intensity (i.e., the standard sound). Another 10% of the sounds are tones of another frequency (1000 Hz) to which the patient responds to by pressing a button (i.e., the target sound). The remaining 10% of sounds are environmental sounds (i.e., a telephone ring, dog bark, etc., the novel sound). The Oddball task takes ~12 min to complete with a total of 320 trials. For all tasks, EEG channels are sampled at 250 Hz.

The BNA assessment utilized a standard high-density 128-­electrode wet EEG cap fitted to the participant’s head. For the Oddball task data, epoched segments (200 ms prestimulus to 1200 ms poststimulus) baseline corrected using the 200 ms prestimulus baseline segments were averaged separately for the “frequent/standard,” “target,” and “novel” sounds. The EEG signals were recorded and cleaned by standard procedures, band-pass filtered into overlapping conventional EEG frequency bands: delta (0.5–4 Hz), theta (3–8 Hz), alpha (7–13 Hz), and beta (12–30 Hz) cut into epochs demarcating pre- and poststimulus onset times, and averaged to align with ERPs. These data were reduced into a set of discrete points that denote local extrema for each band, and the latencies and amplitudes were input to the BNA algorithm.

**Figure 2:**
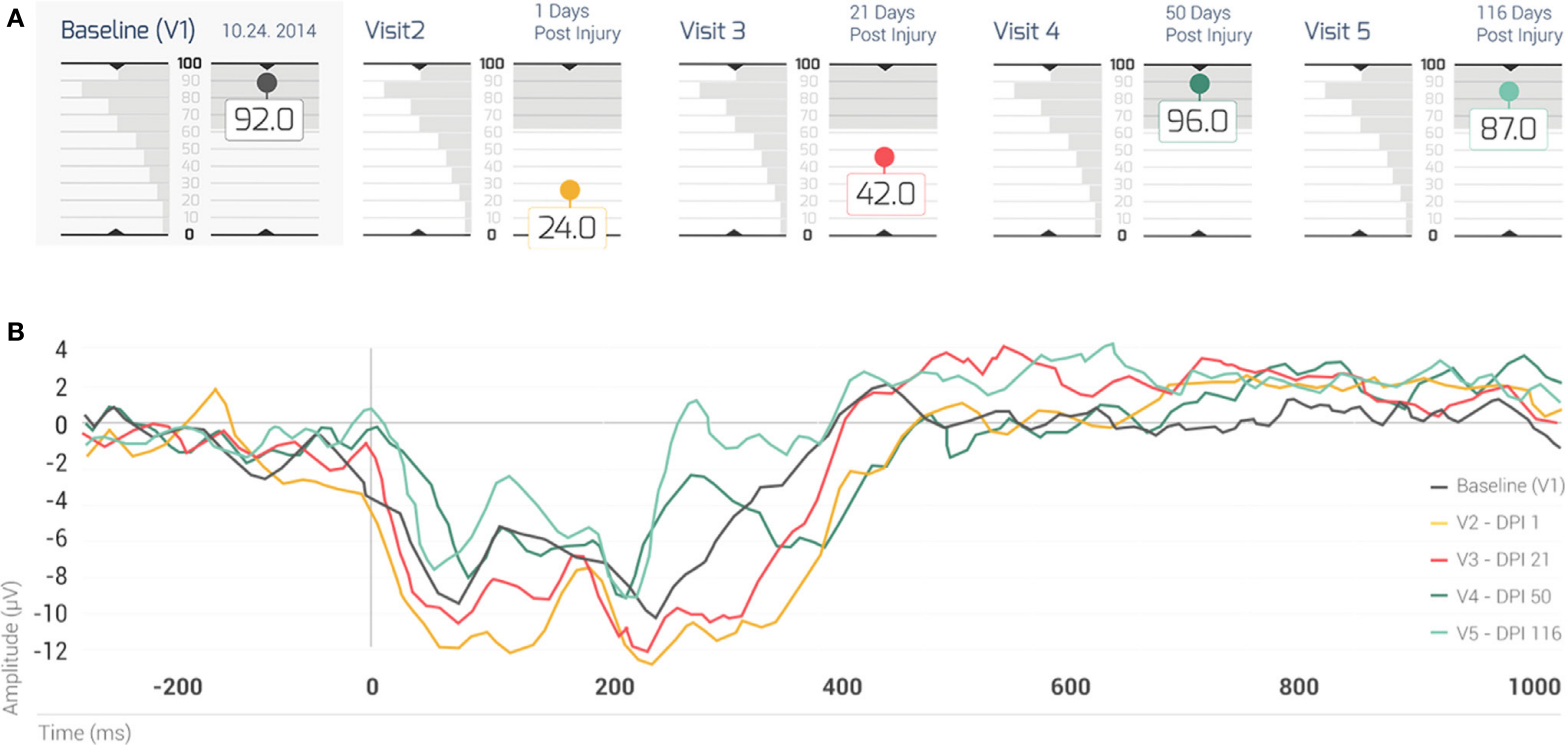
**(A)** The BNA scores of the case for his five visits. The shaded box indicates the test–retest 1 SD of the change between two visits, for a group of 41 normal healthy subjects (i.e., the normal range of change). Note that his BNA scores for visits 2 and 3 (1 and 21 days post concussion, respectively) fall outside of the normal range of change. Each visit is color coded: baseline (visit 1): black; visit 2: brown; visit 3: red; visit 4: dark green; and visit 5: light green. **(B)** The case’s ERP grand average waveforms recorded at the AFz channel. Note that each waveform is associated with a different visit (visits 1–5, respectively) and that the waveforms were color coded in the same manner as the visits in (TOP). EDI = elapsed days from injury; V = visit.

The present case assessed the patient prospectively at 36 h prior to the initial incidence of brain injury, and retrospectively of the injury within 18 h, 21, 50, and 116 days (Figure 2A). The landmark at day 3, which began the progression to RTP, was not part of the study itself and neither was the final clinical visit that cleared for full RTP. The five testing sessions illustrate degrees of accordance between the activation network of the patient at each time point when compared against the RBNM generated for 120 healthy participants between 14 and 16 years of age. At visit one prior to the concussion injury, the Case exhibited a BNA score of 92. Thus, the BNA score measured at baseline computed against a novel negative shift biomarker, indicates typical neural network activation prior to injury. At visit two 18 h post concussion, the Case exhibits a precipitous drop in BNA similarity score to 24, well outside of the typical range of random variation in the BNA score based on the test–retest SD. At 21 days post concussion, the Case’s BNA amplitude increased to 42, but still remained outside of the range defined by the test–retest SD. The Case was symptom free and cleared for RTP progression 5 days after the initial concussion. Despite being symptom free, his neurophysiological data demonstrates significant deficits in the brain network connectivity up to 21 days after the initial injury. It is only after the 3-week follow-up (at both 50 and 116 days post concussion) that the Case’s BNA amplitudes settled back down into the normal range of function (96 and 87, respectively) and matched his pre-injury patterns of activation.

Figure 2B displays the Case’s grand average ERP waveforms recorded at the AFz channel. In general, the first two visits following injury (visits 2 and 3), displayed the most negative ERPs (~180–420 ms) compared with subsequent visits (4 and 5). A gradation could be observed in the level of negativity of the ERP waveforms (Figure 2B) such that the waveform associated with the most enhanced negativity (i.e., the brown ERP trace; Figure 2B) corresponded to the lowest BNA score (visit 2; Figure 2A, brown circle), followed by the negativity at v3 (red trace/red circle, Figures 2A,B, respectively). The other two visits (visits 4 and 5, Figure 2B, dark and light green traces, respectively) in which the BNA score returned to baseline levels (Figure 2A) were associated with less negative ERP waveforms. These two waveforms were clustered together with the baseline trace (Figure 2B, black) above the traces of visits 2 and 3 (Figure 2B). Overall, the changes in the level of negativity displayed by the ERP waveforms were consistent with the recovery trajectory exemplified by the BNA scores (Figures 2A,B).

## Discussion

The present case study is the first of its kind to prospectively and objectively measure the network activation of an adolescent athlete using a BNA-based ERP analysis at 36 h prior to initial incidence of brain injury, and with subsequent follow-up testing within 18 h, 21, 50, and 116 days of the initial injury. The results of this case highlight the potential use of BNA analysis as a clinical tool for evaluating functional network changes following brain injury. BNA analysis may be useful for longitudinal patient monitoring and can help clinicians follow the evolution of suspected cognitive malfunction over time. The head injury sustained by the Case caused functional network changes reflected by a low BNA score in the first follow-up visit, which reflects a reduced similarity to the RBNM of the negative shift immediately following injury (Figure 2). It is important to note that this does not necessarily coincide with less activation in the patient’s brain. It only means that the topography of the patient’s BNA exhibited greater differences from the RBNM. The negative shift observed in the grand average waveform of the group of adolescents (Figure 2) that was used to generate the RBNM may be related to the rapid cognitive growth periods evident in the age range of 14–17 years (18, 29). These rapid growth periods may be associated with continuing maturation of basic sound detection processes, such as changes in the cytoarchitecture, maturation of synaptic efficacy, and continuing myelinization (30). Moreover, a related phenomenon of age-related reduction in slow-wave power parallels the age-related reduction in gray matter volume especially in association cortices, which undergo a significant change in adolescence and early adulthood (31, 32).

Additionally, the results of the case study presented here are in accord with findings in the literature indicating that the adolescent age group is particularly vulnerable to the effects of concussion on the frontal cortex: an area that plays a key role in executive function (33). Since this area of the cortex undergoes continuing developmental changes during adolescence, the vulnerability of the adolescent age group to the effects of concussion might be explained by the enhanced susceptibility to these effects during this time period (3, 34). Compared to network analyses based on functional imaging techniques [such as functional magnetic resonance imaging (fMRI) and positron emission tomography (PET)], EEG-based methods, such as the one presented here, lack the ability to directly sample activity in sub-cortical regions. Nevertheless, the literature indicates the implication of the prefrontal cortex in oddball-related activation (35). Taken together, it may be assumed that the reduction in the BNA score that occurred immediately following injury (i.e., visits 2 and 3; Figure 2) may be related to the greater susceptibility of adolescents to the effects of concussion due to the maturational processes described above.

## Conclusion

This case study demonstrates a potential dissociation between the neurophysiological data and the clinical symptoms associated with concussion. It is possible that this dissociation was enhanced by age-related maturation processes as it was previously found that adolescents exhibited prolonged neurophysiological deficiencies that persisted at least 6 months following a concussion and were more vulnerable than either adults or children to the effects of concussion (12). Thus, the reported case highlights the potential benefits of an objective, sensitive, and aged-based assessment tool to inform clinical management of concussion as well as evaluate the associated changes in the underlying brain networks. The BNA assessment utilized in this case study may be an example of such an analytic tool since it can both qualitatively describe and quantitatively assess the network dynamics associated with brain injury. Additionally, this case also emphasizes the need for care providers to consider both electrophysiological along with clinical indications when managing patient recovery. The current case supports the use of BNA analysis as a supportive clinical tool for clinicians to evaluate electrophysiological network changes associated with sports-related mild traumatic brain injury and monitoring the subsequent recovery. Moreover, these data provide promising evidence that such an approach could be a useful step in categorizing the temporal and spatial changes in brain activity, as well as the functional connectivity of brain networks, associated with concussion injuries.

## Conflict of Interest Statement

Amit Reches, Michal Weiss, and Ilan Laufer have financial conflicts of interest with ElMindA technology. The remaining authors have no conflict of interest to declare.

## References

[1] McCrory P Meeuwisse WH Aubry M Cantu B Dvořák J Echemendia RJ Consensus statement on concussion in sport: the 4th International Conference on concussion in sport, Zurich, November 2012. J Athl Train (2013) 48(4):554–75. 10.4085/1062-6050-48.4.05 23855364 PMC3715021

[2] McKeever CK Schatz P. Current issues in the identification, assessment, and management of concussions in sports-related injuries. Appl Neuropsychol (2003) 10(1):4–11. 10.1207/S15324826AN1001_2 12734070

[3] Baillargeon A Lassonde M Leclerc S Ellemberg D. Neuropsychological and neurophysiological assessment of sport concussion in children, adolescents and adults. Brain Inj (2012) 26(3):211–20. 10.3109/02699052.2012.654590 22372409

[4] Moore RD Hillman CH Broglio SP. The persistent influence of concussive injuries on cognitive control and neuroelectric function. J Athl Train (2014) 49(1):24–35. 10.4085/1062-6050-49.1.01 24377962 PMC3917292

[5] Moore RD Broglio SP Hillman CH. Sport-related concussion and sensory function in young adults. J Athl Train (2014) 49(1):36–41. 10.4085/1062-6050-49.1.02 24377961 PMC3917293

[6] Marar M McIlvain NM Fields SK Comstock RD. Epidemiology of concussions among United States high school athletes in 20 sports. Am J Sports Med (2012) 40(4):747–55. 10.1177/0363546511435626 22287642

[7] Rosenthal JA Foraker RE Collins CL Comstock RD. National high school athlete concussion rates from 2005-2006 to 2011-2012. Am J Sports Med (2014) 42(7):1710–5. 10.1177/0363546514530091 24739186

[8] Mez J Stern RA McKee AC. Chronic traumatic encephalopathy: where are we and where are we going? Curr Neurol Neurosci Rep (2013) 13(12):407. 10.1007/s11910-013-0407-7 24136455 PMC4550089

[9] Echemendia RJ Julian LJ. Mild traumatic brain injury in sports: neuropsychology’s contribution to a developing field. Neuropsychol Rev (2001) 11(2):69–88. 10.1023/A:1016651217141 11572472

[10] Grubenhoff JA Kirkwood MW Deakyne S Wathen J. Detailed concussion symptom analysis in a paediatric ED population. Brain Inj (2011) 25(10):943–9. 10.3109/02699052.2011.597043 21749192

[11] Kutcher JS McCrory P Davis G Ptito A Meeuwisse WH Broglio SP. What evidence exists for new strategies or technologies in the diagnosis of sports concussion and assessment of recovery? Br J Sports Med (2013) 47(5):299–303. 10.1136/bjsports-2013-092257 23479488

[12] Barr WB Prichep LS Chabot R Powell MR McCrea M. Measuring brain electrical activity to track recovery from sport-related concussion. Brain Inj (2012) 26(1):58–66. 10.3109/02699052.2011.608216 22107157

[13] McCrory P Meeuwisse WH Echemendia RJ Iverson GL Dvorák J Kutcher JS. What is the lowest threshold to make a diagnosis of concussion? Br J Sports Med (2013) 47(5):268–71. 10.1136/bjsports-2013-092247 23479483

[14] Mizrahi EM Kellaway P. Cerebral concussion in children: assessment of injury by electroencephalography. Pediatrics (1984) 73(4):419–25. 6709421

[15] Pointinger H Sarahrudi K Poeschl G Munk P. Electroencephalography in primary diagnosis of mild head trauma. Brain Inj (2002) 16(9):799–805. 10.1080/02699050210131911 12217205

[16] Theriault M De Beaumont L Gosselin N Filipinni M Lassonde M. Electrophysiological abnormalities in well functioning multiple concussed athletes. Brain Inj (2009) 23(11):899–906. 10.1080/02699050903283189 20100126

[17] Rapp PE Keyser DO Albano A Hernandez R Gibson DB Zambon RA Traumatic brain injury detection using electrophysiological methods. Front Hum Neurosci (2015) 9:11. 10.3389/fnhum.2015.00011 25698950 PMC4316720

[18] Slobounov S Gay M Johnson B Zhang K. Concussion in athletics: ongoing clinical and brain imaging research controversies. Brain Imaging Behav (2012) 6(2):224–43. 10.1007/s11682-012-9167-2 22669496

[19] Virji-Babul N Hilderman CG Makan N Liu A Smith-Forrester J Franks C Changes in functional brain networks following sports-related concussion in adolescents. J Neurotrauma (2014) 31(23):1914–9. 10.1089/neu.2014.3450 24956041

[20] Keightley ML Chen J-K Ptito A. Examining the neural impact of pediatric concussion: a scoping review of multimodal and integrative approaches using functional and structural MRI techniques. Curr Opin Pediatr (2012) 24(6):709–16. 10.1097/MOP.0b013e3283599a55 23080128

[21] Semple BD Lee S Sadjadi R Fritz N Carlson J Griep C Repetitive concussions in adolescent athletes – translating clinical and experimental research into perspectives on rehabilitation strategies. Front Neurol (2015) 6:69. 10.3389/fneur.2015.00069 25883586 PMC4382966

[22] Karlin AM. Concussion in the pediatric and adolescent population: “different population, different concerns”. PM R (2011) 3(10 Suppl 2):S369–79. 10.1016/j.pmrj.2011.07.015 22035679

[23] Shahaf G Reches A Pinchuk N Fisher T Ben Bashat G Kanter A Introducing a novel approach of network oriented analysis of ERPs, demonstrated on adult attention deficit hyperactivity disorder. Clin Neurophysiol (2012) 123(8):1568–80. 10.1016/j.clinph.2011.12.010 22261156

[24] Reches A Kerem D Gal N A novel ERP pattern analysis method for revealing invariant reference brain network model. Funct Neurol Rehabil Ergon (2013) 3:295–317.

[25] Reches A Laufer I Ziv K Cukierman G McEvoy K Ettinger M Network dynamics predict improvement in working memory performance following donepezil administration in healthy young adults. Neuroimage (2013) 88C:228–41. 10.1016/j.neuroimage.2013.11.020 24269569 PMC4096443

[26] Reches A Levy-Cooperman N. Brain Network Activation (BNA) reveals scopolamine-induced impairment of visual working memory. J Mol Neurosci (2014) 54:59–70. 10.1007/s12031-014-0250-6 24535560

[27] Reches A Nir RR Shram MJ Dickman D Laufer I Shani-Hershkovich R A novel electroencephalography-based tool for objective assessment of network dynamics activated by nociceptive stimuli. J Pain. 10.1002/ejp.716 25960035

[28] Polich J P300 in clinical applications. In: Niedermeyer E de Silva F, editors. Electroencephalography, Basic Principles, Clinical Applications, and Related Fields. Baltimore, MD: Urban and Schwarzenberg (1999). p. 1073–91.

[29] Daniel JC Olesniewicz MH Reeves DL Tam D Bleiberg J Thatcher R Repeated measures of cognitive processing efficiency in adolescent athletes: implications for monitoring recovery from concussion. Neuropsychiatry Neuropsychol Behav Neurol (1999) 12(3):167–9. 10456800

[30] Sussman E Steinschneider M Gumenyuk V Grushko J Lawson K. The maturation of human evoked brain potentials to sounds presented at different stimulus rates. Hear Res (2008) 236(1–2):61–79. 10.1016/j.heares.2007.12.001 18207681 PMC2567844

[31] Whitford TJ Rennie CJ Grieve SM Clark CR Gordon E Williams LM. Brain maturation in adolescence: concurrent changes in neuroanatomy and neurophysiology. Hum Brain Mapp (2007) 28(3):228–37. 10.1002/hbm.20273 16767769 PMC6871488

[32] Mahajan Y McArthur G. Maturation of auditory event-related potentials across adolescence. Hear Res (2012) 294(1–2):82–94. 10.1016/j.heares.2012.10.005 23103362

[33] Howell D Osternig L Van Donkelaar P Mayr U Chou L-S. Effects of concussion on attention and executive function in adolescents. Med Sci Sports Exerc (2013) 45(6):1030–7. 10.1249/MSS.0b013e3182814595 23274602

[34] Luna B Garver KE Urban TA Lazar NA Sweeney JA. Maturation of cognitive processes from late childhood to adulthood. Child Dev (2004) 75(5):1357–72. 10.1111/j.1467-8624.2004.00745.x 15369519

[35] Huettel SA Mccarthy G. What is odd in the oddball task? Prefrontal cortex is activated by dynamic changes in response strategy. Neuropsychologia (2004) 42(3):379–86. 10.1016/j.neuropsychologia.2003.07.009 14670576

